# *Staphylococcus aureus* Clinical Isolates: Antibiotic Susceptibility, Molecular Characteristics, and Ability to Form Biofilm

**DOI:** 10.1155/2013/314654

**Published:** 2013-08-31

**Authors:** N. Indrawattana, O. Sungkhachat, N. Sookrung, M. Chongsa-nguan, A. Tungtrongchitr, S. P. Voravuthikunchai, T. Kong-ngoen, H. Kurazono, W. Chaicumpa

**Affiliations:** ^1^Department of Microbiology and Immunology, Faculty of Tropical Medicine, Mahidol University, Bangkok 10400, Thailand; ^2^Department of Research and Development, Faculty of Medicine Siriraj Hospital, Mahidol University, Bangkok 10700, Thailand; ^3^Department of Parasitology, Faculty of Medicine Siriraj Hospital, Mahidol University, Bangkok 10700, Thailand; ^4^Natural Products Research Center and Department of Microbiology, Faculty of Science, Prince of Songkla University, Songkhla 90112, Thailand; ^5^Department of Applied Veterinary Medicine and Public Health, Obihiro University of Agriculture and Veterinary Medicine, Inada-cho, Obihiro, Hokkaido 080 8555, Japan

## Abstract

Periodic monitoring of *Staphylococcus aureus* characteristics in a locality is imperative as their drug-resistant variants cause treatment problem. In this study, antibiograms, prevalence of toxin genes (*sea-see, seg-ser, seu, tsst-1, eta, etb*, and *etd*), PFGE types, accessory gene regulator (*agr*) groups, and ability to form biofilm of 92 *S. aureus* Thailand clinical isolates were investigated. They were classified into 10 drug groups: groups 1–7 (56 isolates) were methicillin resistant (MRSA) and 8–10 (36 isolates) were methicillin sensitive (MSSA). One isolate did not have any toxin gene, 4 isolates carried one toxin gene (*seq*), and 87 isolates had two or more toxin genes. No isolate had *see, etb*, or *tsst-1*; six isolates had *eta* or *etd*. Combined *seg-sei-sem-sen-seo* of the highly prevalent *egc* locus was 26.1%. The *seb, sec, sel, seu*, and *eta* associated significantly with MSSA; *sek* was more in MRSA. The *sek-seq* association was 52.17% while combined *sed-sej* was not found. Twenty-three PFGE types were revealed, no association of toxin genes with PFGE types. All four *agr* groups were present; *agr* group 1 was predominant (58.70%) but *agr* group 2 strains carried more toxin genes and were more frequent toxin producers. Biofilm formation was found in 72.83% of the isolates but there was no association with antibiograms. This study provides insight information on molecular and phenotypic markers of Thailand *S. aureus* clinical isolates which should be useful for future active surveillance that aimed to control a spread of existing antimicrobial resistant bacteria and early recognition of a newly emerged variant.

## 1. Introduction

*Staphylococcus aureus*, a gram positive coccal bacterium, is either commensal that colonizes healthy nasal mucosa [1] or pathogen of humans. As a pathogen, the bacteria cause a variety of community and hospital acquired diseases including skin abscess [2], food poisoning [3], pneumonitis [4], sepsis [5], and toxic shock syndrome [6]. This bacterium produces several virulent factors including adhesins (colonization factors), toxic proteins/enzymes (e.g., DNase for bacterial spread, coagulase, and catalase for host immunity evasion) and exotoxins including exfoliative toxins (ExTs), staphylococcal enterotoxins (SEs), and toxic shock syndrome toxin-1 (TSST-1). Patients infected with the ExT producing *S. aureus* may develop scalded-skin syndrome [7]. The SEs and TSST-1, besides causing food poisoning, are also superantigens (SAg) that can stimulate a relatively large fraction of peripheral blood T cells to release massive amounts of proinflammatory cytokines and T-cell stimulating factors leading to toxic shock syndrome which may be fatal [8, 9]. The enterotoxicity and superantigenicity are distinct properties of the toxin molecule [6]. SEs are classified into two types based on their emetic activity in the toxin fed modeled primate. Toxins that induce vomiting in the primate are placed in the classical SE type while those that lack the emetic activity or have not been tested are allocated in the SE-like (SE*l*s) type [10, 11]. Members of the classical SEs are SEA-SEE and the more recently recognized SEG, SEH, SEI, SER, SES, and SET. The SE*l*s members include SE*l*J, SE*l*K, SE*l*L, SE*l*M, SE*l*N, SE*l*O, SE*l*P, SE*l*Q, SE*l*U, SE*l*U2 or SEW, and SE*l*V [11]. The staphylococcal enterotoxin F (SEF) which lacks emetic activity but is associated with toxic shock syndrome is presently called toxic shock syndrome toxin-1 (TSST-1) [12]. The SEs and the TSST-1 as well as the bacterial resistance to drugs are encoded by genes on the mobile genetic elements including prophages, plasmids, pathogenicity islands, genomic islands, and antibiotic resistance cassette [13]; thus they are transmitted horizontally rather easily. Expression of *S. aureus* virulence factors and metabolism of metabolic pathways during growth are coordinated/regulated by a quorum-sensing operon named accessory gene regulator (*agr*) [14, 15]. Based on the amino acid sequence polymorphisms of the *agr*-encoding autoinducing peptides and their responding receptors, *S. aureus* strains can be divided into four major *agr* groups (groups 1–4) [16].

 During the last five decades, *S. aureus* clones that resist methicillin (methicillin-resistant *S. aureus*, MRSA) disseminated and caused medical and public health problem worldwide [17, 18]. These strains are not only resistant to methicillin, but also resistant to all other *β-*lactams, such as cephalosporin [18, 19]. In Thailand, MRSA infections were reported from 23 hospitals from 1988 to 1998 [20, 21]. The proportions of MRSA to MSSA in the northeast, central, and southern regions of the country during the studied period increased from 11 to 23.4%, 16 to 30.5%, and 21 to 30.3%, respectively [22]. Moreover, methicillin-resistant *S. aureus* with reduced susceptibility to vancomycin was recognized [23]. However, data on genotypic characteristics and other attributes of the *S. aureus* isolates in Thailand are relatively rare. Therefore, this study investigated the prevalence of virulence toxin genes coding for enterotoxins (*sea-see, seg-ser*, and *seu*), toxic shock syndrome toxin-1 (*tsst-1*), and exfoliative toxins (*eta*,* etb,* and *etd*) among *S. aureus* Thailand clinical isolates. Molecular diversity of the isolates regarding their endonuclease-restricted patterns of genomic DNA (PFGE),* agr* types, and antimicrobial susceptibility as well as their ability to produce biofilm were also investigated.

## 2. Materials and Methods 

### 2.1. Bacterial Strains

Ninety-two strains of *S. aureus* isolated from clinical specimens were obtained from three hospitals. They were 43 strains (S1–S43) isolated in 2007 from patients of Prince of Songkla University Teaching Hospital and kept at the Department of Microbiology, Faculty of Science, Prince of Songkla University, Songkhla province, southern Thailand; 36 strains (P1–P36) from the patients of Prasat Neurological Institute, Bangkok, in 2010, and 13 strains (T1–T13) isolated in 2010 from patients of the Hospital for Tropical Diseases, Faculty of Tropical Medicine, Mahidol University, Bangkok, Thailand. The bacteria were reconfirmed by Gram staining, biochemical testing (catalase, coagulase, and DNase), and mannitol fermentation. Their ability to produce protein A was detected by agglutination assay. 

### 2.2. Antimicrobial Susceptibility Testing

Disc diffusion method was used for antimicrobial susceptibility testing of the *S. aureus* isolates which was done according to CLSI guidelines [24]. Antibiotic discs were cefoxitin, ciprofloxacin, clindamycin, erythromycin, gentamycin, oxacillin, penicillin G, rifampin, tetracycline, sulfamethoxazole plus trimethoprim, and teicoplanin (Oxoid, UK). Cefoxitin disc (30 *μ*g) and oxacillin disc (1 *μ*g) were used for detecting methicillin-resistant isolates. *S. aureus* ATCC 25923 was used as control. Reduction of vancomycin susceptibility of the isolates was also determined by observing the minimum inhibitory concentration (MIC) by agar dilution according to the CLSI guidelines [24].

### 2.3. Detection of Genes Coding for Staphylococcal Enterotoxins, TSST-1, and ExTs

Genomic DNA was extracted from each *S. aureus* isolate by DNA extraction kit (Geneaid, Taiwan) following the protocol for Gram-positive bacteria. Quality of each DNA preparation was assessed by determining the ratio of OD_260 nm_/OD_280 nm_. Twenty-two virulence genes were amplified including *sea-see*, *seg-ser* and* seu*, *tsst-1* and *eta*, *etb* and *etd*, using specific oligonucleotide primer sequences listed in Table 1 [25, 26]. The PCR reaction mixture (25 *μ*L) is composed of 1 mM of each primer, 1x *Taq* buffer PCR, 0.2 mM dNTP, 2 mM MgCl_2_, 1 unit of *Taq* DNA polymerase (Fermentas, Germany), and 100 ng of DNA template. The PCR reaction mixture was subjected to the thermal cycles: an initial denaturation of DNA at 95°C for 10 min prior to 35 cycles of denaturation at 95°C for 30 sec, 55°C for 30 sec, and 72°C for 30 sec, followed by a final extension of 10 min at 72°C using the Lifecycler (BioRad, USA). The amplified products were analyzed by 1.5% agarose gel electrophoresis and ethidium bromide staining. The DNA bands were observed under an UV transilluminator (Syngene, England). Control bacteria for the PCR included strains ATCC 19095 (*sea, sec*, *seh*, *seg*, *sei*, *sel*, *sem*, *sen*, *seo*, *seu,* and *tst*), ATCC 14458 (*seb* and *sek*), ATCC 23235 (*sed*, *sej*), and ATCC 27664 (*see*, *seq,* and *sea*). For *eta*, *etb,* and *etd*, the PCR amplicons were verified by DNA sequencing and the nucleotide sequences were aligned with the staphylococcal *eta, etb,* and *etd* sequences of the database (accession numbers: L25372.1, M17348.1, and AB057421.1, resp.).

### 2.4. Detection of SEs, TSST-1, and ExTs

The bacterial isolates which carried *sea, seb, sec *and* sed; eta *and* etb*; *tsst-1* were tested for their ability to express the respective proteins by the reversed-passive latex agglutination (RPLA) using commercially available kits: SET-RPLA, TST-RPLA, and EXT-RPLA (Denka Seiken, Japan), respectively. Other toxin detections were not done due to lack of available test kits.

### 2.5. Pulsed-Field Gel Electrophoresis (PFGE)

PFGE patterns of chromosomal DNA of all *S. aureus* isolates were determined by digesting each DNA preparation with *Sma*I. The digested DNA preparations were subjected to electrophoretic separation in a CHEF-DR II system (BioRad, USA) as described previously [27]. DNA fragment patterns were analyzed in the GeneDirectory Application Version 2.01.00 Copyright 2000–2008 Synoptics Ltd. Percent similarities were identified on dendrogram derived from the unweighted pair group method with arithmetic averages (UPGMA) and based on Dice coefficients. Band position tolerance was set at 1.0%. A coefficient similarity of 70% was selected to define cluster of the PFGE types.

### 2.6. The *Agr* Alleles

Genomic DNA of the 92 *S. aureus* isolates was used as templates for amplification of *agr* alleles using the group specific primers [16]. The common forward (pan) primer: (5′-ATGCACATGGTGCACATGC-3′) and reversed primers including: agr1 (5′-GTCACAAGTACTATAAGCTGCGAT-3′), agr2 (5′-TATTACTAATTGAAAAGTGCCATAGC-3′), agr3 (5′-GTAATGTAATAGCTTGTATAATAATACCCAG-3′), and agr4 (5′-CGATAATGCCGTAATACCCG-3′) were used. These primers allowed amplification of 439-, 572-, 320-, and 657-bp DNA fragments of the *agr* groups 1–4, respectively.

### 2.7. Biofilm Formation

Ability of the *S. aureus* isolates to form biofilm was determined according to the protocol described previously [28] with modification. Individual bacterial isolates were cultured in TSB (Oxoid) supplemented with 0.25% glucose at 35°C until the turbidity reached McFarland no. 0.5. Approximately 100 cfu of each culture were applied in triplicate into wells of 96-well flat-bottomed microplate containing 200 *μ*L of the TSB and 0.25% glucose. Wells added with cultured *S. epidermidis* (ATCC12228) served as negative controls. The plate was incubated for 24 h. The content of each well was then discarded and the wells were washed five times with sterile 0.9% NaCl solution. Each well surface was stained by adding 100 *μ*L of 0.3% (w/v) crystal violet (Merck) in water and kept for 5 min. After five washing with sterile distilled water and air dried. The biofilm fixed on each well surface was extracted with 100 *μ*L of 70% ethanol and measured the absorbance at OD_570 nm_. The isolates with OD_570 nm_ values above the mean OD_570 nm_ values plus three standard deviations of the negative control (mean_neg_ + 3 SD_Neg_) were considered positive for biofilm formation. 

### 2.8. Statistical Analyses

SPSS Statistics 16.0 was used for statistical analysis. Chi-squared (**χ** ^2^) test and *t*-test were used to analyze the data sorted by MRSA and MSSA groups and frequencies of virulence genes and biofilm formation, respectively. A probability value (*P*) < 0.05 was considered different significantly.

## 3. Results

### 3.1. Antimicrobial Susceptibility

All of the 92 bacterial isolates from culture stocks were verified as *S. aureus* strains according to their phenotypic characteristics determined by the conventional microbiological method. After testing with the 30 *μ*g cefoxitin disc, 56/92 isolates (60.87%) were MRSA (37 isolates from the Prince of Songkla hospital and 19 isolates from Prasat Neurological Institute), and 36 isolates (39.13%) were MSSA (5 isolates from the Prince of Songkla hospital, 17 isolates from the Prasat Neurological Institute, and 19 isolates from the Hospital for Tropical Diseases). The 92 *S. aureus* Thailand isolates were arbitrarily classified into 10 drug groups. Groups 1–7 were MRSA and groups 8–10 were MSSA. Data on susceptible and intermediate sensitivity to the 11 antibiotics tested (cefoxitin, ciprofloxacin, clindamycin, erythromycin, gentamicin, oxacillin, penicillin, rifampin, trimethoprim/sulfamethoxazole (T/S), tetracycline, and teicoplanin) were group 1 (16 isolates): susceptible (9 isolates) and intermediate (7 isolates) to rifampin and susceptible to teicoplanin; group 2 (2 isolates): susceptible to gentamicin and teicoplanin, intermediate to rifampin; group 3 (7 isolates): susceptible to gentamicin and teicoplanin; group 4 (1 isolate): susceptible to tetracycline and teicoplanin; group 5 (7 isolates): susceptible to rifampin, trimethoprim/sulfametoxazole, tetracycline, and teicoplanin and susceptible to gentamicin (1 isolate); group 6 (10 isolates): susceptible to rifampin, trimethoprim/sulfametoxazole (10 isolates), intermediate to trimethoprim/sulfametoxazole (1 isolate), and susceptible to teicoplanin; group 7 (13 isolates): susceptible to teicoplanin; group 8 (3 isolates): susceptible to oxacillin (2 isolates), cefoxitin, gentamicin, gentamicin, and teicoplanin; group 9 (28 isolates): resistant to penicillin and tetracycline (13 isolates), intermediate to erythromycin (1 islates); group 10 (5 isolates): resistant to gentamicin (1 isolate), ciprofloxacin (2 isolates), erythromycin (2 isolates), and clindamycin (2 isolates). All of the isolates were sensitive to vancomycin according to the MIC testing. The methicillin susceptibility and drug groups of the 92 isolates are shown in Table 2.

### 3.2. Prevalence of Toxin Genes in Individual *S. aureus* Isolates

**Figure 1:**
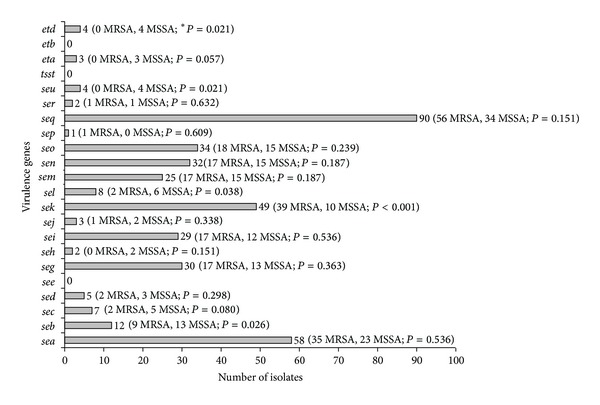
Prevalence of the enterotoxin and exfoliative toxin genes among the 92 *S. aureus* Thailand isolates. *P value between prevalence of MRSA compared to MSSA.

Among the 92 isolates, 1 isolate (1.08%) did not have any toxin gene (S38), 4 (4.35%) isolates (S16, S33, S40, and P33) carried one toxin gene (*seq*), and the remaining 87 isolates (94.57%) carried two or more toxin genes (Table 2). There were only 6/92 isolates that carry the *etx *genes either *eta* or *etd* (P28, P31, T3, T8, T9, and T13). The prevalence of toxin genes among the isolates is shown in Figure 1. The predominant enterotoxin gene was *seq *which was presented in 91/92 isolates (98.91%), followed by *sea* (65.22%) and *sek* (54.35%). There was no isolate with *see*, *tsst-1* (*sef*), or *etb*. The prevalence of *sea, sec, sed, seg, seh, sei, sej, sem, sen, seo, sep, seq, ser, eta, *and* etd *among the MRSA and MSSA isolates were not different. However, the prevalence of *seb, sel, *and *seu *among isolates of the two methicillin groups was different significantly.

### 3.3. Determination of Toxin Production

The bacterial isolates which carried *sea*, *seb*, *sec*, *sed*; *eta* and *etb*; *tsst-1* were determined for their ability to produce the respective toxins by using SET-RPLA, TST-RPLA, and EXT-RPLA, respectively, and 35 isolates were toxin producers (Table 2). There were 21/60 *sea* strains (35%) that produced SEA; 9/13 *seb* isolates (69.23%) produced SEB; 4/7 *sec* isolates (57.14%) produced SEC; and 3/5 *sed *isolates (60%) produced SED. One of the three *eta *positive strains (33.33%) could produce ETA. None of the four isolates with *etd*-positive strains produced ETD. Among the MRSA, 24/56 isolates (42.86%) produced toxins (17 SEA and 7 SEB), whereas 11/36 (30.55%) of the MSSA isolates produced toxins (SEA 4 isolates, SEB 1 isolate, SEC 3 isolates, SED 2 isolates, and SEB and ETA 1 isolate). There were 3 MSSA isolates that produced more than one toxin: S41 produced SEB and SED, P23 produced SEA and SEC, and T3 produced SEB and ETA. 

### 3.4. PFGE Types

**Figure 2:**
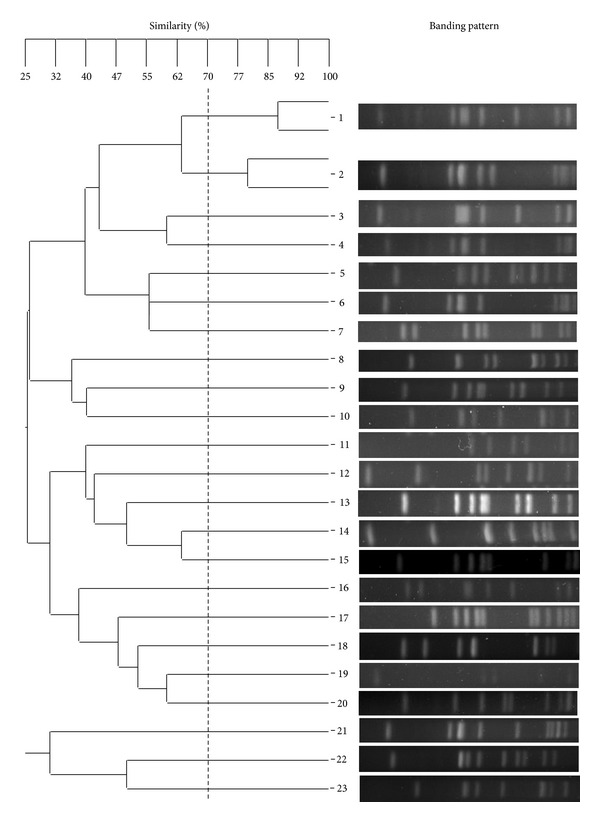
Dendrogram of PFGE patterns the 92 *S. aureus* Thailand isolates.

The 92 *S. aureus* isolates could be classified according to the PFGE results into 23 genotypes, genotypes 1–23 (Figure 2). Information on the PFGE types of individual isolates is given in Table 2. PFGE type 21 was predominant (16 isolates), followed by types 1, 9, and 22 (13, 11, and 10 isolates, resp.); types 2 and 18 had 6 isolates each; types 4 and 6 had 4 isolates each; 3 isolates belonged to type 15; types 5, 8, 17, 20, and 23 had 2 isolates each, and types 3, 7, 10, 11, 12, 13, 14, 16, and 19 had 1 isolate each. 

### 3.5. The *Agr* Groups

The predominant *agr* group among the 92 isolates was group 1 (54/92 isolates; 58.70%) followed by groups 2 (29 isolates; 31.52%), 3 (6 isolates; 6.52%), and 4 (3 isolates; 3.26%). 

### 3.6. Biofilm Formation

There were 67/92 isolates (72.83%) that produced biofilm; 21/36 (58.33%) were MSSA and 46/56 isolates (82.14%) were MRSA. The prevalence of the biofilm formation of the MRSA and MSSA was not different (*P* > 0.05).

## 4. Discussion

Diseases caused by *S. aureus* are health hazard to human worldwide. Since the first recognition of methicillin-resistant *S. aureus* in 1961 [29], there has been an upsurge of infections caused by the *S. aureus* variants that resist not only methicillin, but also other *β*-lactams and vancomycin, which are therapeutic drugs of choice [30–32], leading to treatment failure and increased case fatality rate. The methicillin and vancomycin resistance of the *S. aureus *are encoded by staphylococcal cassette chromosome *mec* (SCC*mec*) and *vanA*, respectively [30, 31]. Association of the presence of *S. aureus* toxin genes with methicillin sensitivity and resistance among *S. aureus* has been reported previously [28, 33–35]. The association was found also in the present study; the prevalence of the *seb, sec, sel, seu,* and *eta *was associated significantly (*P* < 0.05) with the MSSA while *sek* was found more in MRSA. 

 The toxin genes carried by the 92 Thailand isolates varied from none to as many as 11 genes (Table 2). Five of the *S. aureus* enterotoxin genes, that is, *seg, sei, sem, sen,* and *seo*, belonged to the highly prevalent *egc* locus [36, 37]; thus, their coexistence was frequently reported. Coexistence of *seg*-*sei* in the same strain, either alone or in more combination with other toxin gene(s) (*sea*, *sec*, *sed*, *seh*, *sej,* and/or *tst*) was found in 55% of the 429 *S. aureus* isolates from Germany [38]. In Japan, the *seg-sei* alone or with *seb, sec,* or *sed* were 24, 2.7, 6.8, and 2.0%, respectively [39]. The combined *seg*-*sei*-*sem*-*sen*-*seo* with *seu* was 15.1% among the Chinese isolates [26]. In the present study, the combined *seg*-*sei-sem-sen-seo* with other toxin genes including *sea, seb, sed, sej, sek, sel, sep, seq, ser, *and/or *eta* was found in 24/92 isolates (26.1%). There were 3 isolates that carried *seg-sei-sen-seo* with *sea, sec, sek, sel,* and/or *seq* and 1 isolate with *seg-sei-sem-sen *and* seb*. The previously reported fixed association of *sed-sej* [38] was not found among the 92 Thailand isolates. The combined *sek-seq *with other toxin gene(s), that is, *sea* and/or *seb*, was 45.5% among the Chinese isolates [26]. In the present study, the *sek-seq* association was found in 48 of the 92 isolates (52.17%), either the two genes alone (16.3%) or with the other toxin genes (35.86%). 

 The ability of the isolates to produce SEA, SEB, SEC, and SED and ETA, ETB, and TSST-1 was examined by using SET-RPLA, TST-RPLA, and EXT-RPLA test kits, respectively. Not all isolates harboring the genes expressed the respective toxins. The results were similar to the finding reported previously among *S. aureus* isolates from milk and milk products from Morocco [40]. The unconformed results between genotypes (by PCR) to phenotypes (by RPLA) could be due to the fact that toxin production of the bacteria can be affected by the growth conditions including temperature, pH, and water activity. The so-produced toxin levels might be lower than the detection limits of the immunoassay [40, 41]. Alternatively, the toxin gene may not be expressed due to mutation either in the coding region or in a regulatory region, for example, *agr* [42, 43]. No annotated data are available in the literature on association of the ability of toxin production and antibiograms of the *S. aureus.* Nevertheless, in this study, the frequency of toxin production is higher among the MRSA (48.86%) than the MSSA (30.55%) (*P* < 0.05). 

 There was no association between PFGE patterns with the MRSA and MSSA of the 92 Thai strains which conformed to the results reported elsewhere [44, 45]. However, PFGE patterns 21 and 22 of MRSA strains predominated among isolates from Prince of Songkla Hospital and Prasat Neurological Institute, that is, 32.5 and 27.8%, respectively. Among the 7 isolates of PFGE pattern 21 of Songkla that could produce enterotoxins, 6 strains (85.7%) produced SEA. All 7 isolates of PFGE type 22 of Prasat Neurological Institute isolates produced SEB. 

 The polymorphism in the *agr* locus was first described by Ji et al. in 1997 [46]. To date, *S. aureus* isolates were classified into four different *agr *groups [25, 46]. In this study, all *agr *groups were found; large proportion (58.6%) of the isolates was *agr* group 1 which was similar to the data reported previously [16]. Moreover, majority (38/54 isolates, 70%) of the *agr *group 1 were MRSA which conformed also to the previous report [47]. However, it is noteworthy that isolates of the *agr* group 2 in this study carried more number of enterotoxins genes, and most of the toxin producing strains belonged to this *agr* group. The data were different from elsewhere which showed that most toxin producing *S. aureus* strains were either *agr* groups 3 [46] or 4 [48]. 

 Biofilm formation contributes to bacterial pathogenesis and resistance to antibiotics and harsh environment. *S. aureus* isolates did form biofilms [28, 49]. More strains of MSSA produced biofilm compared to MRSA strains [28]. In this study, 72.83% of the *S. aureus *isolates formed biofilm but there was no association with their antibiotic patterns. 

 In conclusion, the results of this study provide insight information on molecular and phenotypic markers of *S. aureus* clinical isolates in Thailand which should be useful for future active surveillance that aimed to control a spread of existing antimicrobial resistant bacteria as well as early recognition of a newly emerged variant. 

## Figures and Tables

**Table 1:** The primer sequences for amplification of the *S. aureus* enterotoxin genes.

Target gene	Primer sequence 5′→ 3′		Size of PCR product (bp)	Reference
*sea *	(F) (R)	GAAAAAAGTCTGAATTGCAGGGAACA CAAATAAATCGTAATTAACCGAAGGTTC	560	[26]
*seb *	(F) (R)	ATTCTATTAAGGACACTAAGTTAGGGA ATCCCGTTTCATAAGGCGAGT	404	[26]
*sec *	(F) (R)	CTTGTATGTATGGAGGAATAACAAAACATG CATATCATACCAAAAAGTATTGCCGT	275	[26]
*sed *	(F) (R)	GAATTAAGTAGTACCGCGCTAAATAATATG GCTGTATTTTTCCTCCGAGAGT	492	[26]
*see *	(F) (R)	CAAAGAAATGCTTTAAGCAATCTTAGGC CACCTTACCGCCAAAGCTG	482	[26]
*seg *	(F) (R)	ACCTGAAAAGCTTCAAGGA CGCCAACGTAATTCCAC	204	[26]
*seh *	(F) (R)	CAATCACATCATATGCGAAAGCAG CATCTACCCAAACATTAGCACC	376	[26]
*sei *	(F) (R)	CTYGAATTTTCAACMGGTAC AGGCAGTCCATCTCCTG-3	461	[26]
*sej *	(F) (R)	TCAGAACTGTTGTTCCGCTAG GAATTTTACCAYCAAAGGTAC	138	[26]
*sek *	(F) (R1) (R2)	ATGCCAGCGCTCAAGGC AGATTCATTTGAAAATTGTAGTTGATTAGCT TGCCAGCGCTCAAGGTG	134	[26]
*sel *	(F) (R)	GCGATGTAGGTCCAGGAAAC CATATATAGTACGAGAGTTAGAACCATA	234	[26]
*sem *	(F) (R)	CTATTAATCTTTGGGTTAATGGAGAAC TTCAGTTTCGACAGTTTTGTTGTCAT	326	[26]
*sen *	(F) (R)	CGTGGCAATTAGACGAGTC GATTGATYTTGATGATTATKAG	474	[26]
*seo *	(F) (R)	AGTTTGTGTAAGAAGTCAAGTGTAGA ATCTTTAAATTCAGCAGATATTCCATCTAAC	180	[26]
*sep *	(F) (R)	GAATTGCAGGGAACTGCT GGCGGTGTCTTTTGAAC	182	[26]
*seq *	(F) (R)	ACCTGAAAAGCTTCAAGGA CGCCAACGTAATTCCAC	204	[26]
*ser *	(F) (R)	AGCGGTAATAGCAGAAAATG TCTTGTACCGTAACCGTTTT	363	[26]
*seu *	(F) (R)	AATGGCTCTAAAATTGATGG ATTTGATTTCCATCATGCTC	215	[26]
*tst *	(F) (R)	TTCACTATTTGTAAAAGTGTCAGACCCACT TACTAATGAATTTTTTTATCGTAAGCCCTT	180	[26]
*eta *	(F) (R)	ACTGTAGGAGCTAGTGCATTTGT TGGATACTTTTGTCTATCTTTTTCATCAAC	190	[26]
*etb *	(F) (R)	CAGATAAAGAGCTTTATACACACATTAC AGTGAACTTATCTTTCTATTGAAAAACACTC	612	[25]
*etd *	(F) (R)	CAAACTATCATGTATCAAGGATGG CCAGAATTTCCCGACTCAG	358	[26]

**Table 2:** Characteristics of the 92 *S. aureus* Thailand isolates.

Isolate no.	Methicillin susceptibility	Drug group	Enterotoxin gene(s)	ExT gene	RPLA toxin	PFGE type	Agr group	Biofilm (OD)
S1	R	1	*sek*, *seq *	—	ND	1	1	+ (0.831)
S2	R	1	*sea*, *sek*, *seo*, *seq *	—	ND	1	1	+ (0.828)
S3	R	1	*sek*, *seq *	—	ND	1	1	+ (0.039)
S4	R	1	*sek*, *seq *	—	ND	1	1	+ (0.181)
S5	R	1	*sea*, *sek*, *seq *	—	—	1	1	+ (0.701)
S6	R	1	*sek*, *seq *	—	ND	2	1	+ (1.566)
S7	R	1	*sea*, *sed*, *sek*, *seq *	—	—	1	2	+ (1.841)
S8	R	1	*sea*, *sek*, *seq *	—	SEA	6	1	+ (1.701)
S9	R	1	*sea*, *sek*, *seq *	—	SEA	21	1	+ (0.996)
S10	R	1	*sea*, *sek*, *seq *	—	SEA	21	1	+ (1.219)
S11	R	1	*sea*, *sek*, *seo*, *seq *	—	SEA	21	1	+ (1.749)
S12	R	1	*sea*, *sek*, *seq *	—	SEA	21	1	+ (1.377)
S13	R	1	*sea*, *sek*, *seq *	—	SEA	21	1	+ (1.687)
S14	R	1	*sea*, *sek*, *seq *	—	SEA	21	1	+ (0.796)
S15	R	2	*sea*, *sek*, *seq *	—	—	1	1	+ (0.097)
S16	R	2	*seq *	—	ND	3	1	+ (0.132)
S17	R	3	*sea*, *sek*, *seq *	—	—	1	1	+ (0.085)
S18	R	3	*sea*, *sek*, *seq *	—	—	4	1	+ (0.230)
S19	R	3	*sea*, *sek*, *seq *	—	—	4	1	+ (0.080)
S20	R	3	*sek*, *seq *	—	ND	6	1	+ (0.417)
S21	R	3	*sek*, *seq *	—	ND	6	1	+ (1.103)
S22	R	3	*sek*, *seq *	—	ND	6	1	+ (1.835)
S23	R	3	*sek*, *seq *	—	ND	21	1	+ (0.097)
S24	R	4	*sea*, *sek*, *seq *	—	—	2	1	+ (0.552)
S25	R	7	*sea*, *sek*, *seq *	—	—	1	1	+ (0.569)
S26	R	7	*sek*, *seq *	—	ND	1	1	+ (1.000)
S27	R	7	*sek*, *seq *	—	ND	1	1	+ (1.155)
S28	R	7	*sea*, *sek*, *seq *	—	—	1	1	+ (0.715)
S29	R	7	*sek*, *seq *	—	ND	2	1	+ (1.061)
S30	R	7	*sek*, *seq *	—	ND	2	1	+ (1.131)
S31	R	7	*sek*, *seq *	—	ND	2	1	+ (0.774)
S32	R	7	*sea*, *sec*, *sek*, *sel*, *seq *	—	—	9	1	+ (1.796)
S33	R	7	*seq *	—	ND	21	1	+ (2.481)
S34	R	7	*sea*, *sec*, *sel*, *seq *	—	—	21	1	+ (1.000)
S35	R	7	*sea*, *sek*, *seq *	—	—	21	1	+ (1.792)
S36	R	7	*sek*, *seq *	—	ND	21	1	+ (1.184)
S37	R	7	*sek*, *seq *	—	ND	21	1	+ (2.332)
S38	S	8		—	ND	4	1	− (−0.052)
S39	S	8	*sej*, *sek*, *seq *	—	ND	4	2	+ (0.367)
S40	S	8	*seq *	—	ND	21	1	+ (0.508)
S41	S	9	*seb*, *sed*, *sej*, *sek*, *seq*, *ser*, *etd *	—	SEB, SED	21	3	+ (0.074)
S42	S	9	*seg*, *sei*, *sem*, *sen*, *seo*, *seq*, *seu *	—	—	19	3	− (−0.007)
S43	S	9	*seg*, *sei*, *sek*, *sem*, *sen*, *seo*, *seq *	—	—	7	2	− (−0.008)

P1	R	1	*sea*, *seq *	—	SEA	21	1	+ (0.317)
P2	R	1	*sea*, *seg*, *sei*, *sek*, *sen*, *seo*, *seq *	—	SEA	9	2	+ (0.700)
P3	R	5	*sea*, *seg*, *sei*, *sem*, *sen*, *seo*, *seq *	—	SEA	9	2	+ (0.098)
P4	R	5	*sea*, *seg*, *sei*, *sem*, *sen*, *seo*, *seq *	—	SEA	9	2	− (−0.194)
P5	R	5	*sea*, *sei*, *sek*, *sen*, *seo*, *seq *	—	SEA	9	2	+ (0.543)
P6	R	5	*sea*, *seg*, *sei*, *sem*, *sen*, *seo*, *seq *	—	SEA	9	2	− (−0.144)
P7	R	5	*sea*, *seg*, *sei*, *sek*, *sem*, *sen*, *seo*, *seq *	—	SEA	9	2	− (−0.095)
P8	R	5	*sea*, *seg*, *sei*, *sem*, *sen*, *seo*, *seq *	—	SEA	13	2	+ (0.451)
P9	R	5	*sea*, *sed*, *seg*, *sei*, *sej*,* sem*, *sen*, *seo*, *sep*,* seq*, *ser *	—	SED	16	2	− (−0.05)
P10	R	6	*sea*, *sek*, *seq *	—	SEA	21	1	+ (0.817)
P11	R	6	*sea*, *seb*, *seg*, *sei*, *sem*, *sen*, *seo*, *seq *	—	SEB	22	2	+ (0.141)
P12	R	6	*seb*, *seg*, *sei*, *sem*, *sen*, *seo*, *seq *	—	SEB	22	2	+ (0.179)
P13	R	6	*seb*, *seg*, *sei*, *sem*, *sen*, *seq *	—	SEB	22	2	− (−0.176)
P14	R	6	*sea*, *seb*, *seg*, *sei*, *sem*, *sen*, *seo*, *seq *	—	—	22	2	+ (0.182)
P15	R	6	*sea*, *seb*, *seg*, *sei*, *sem*,* sen*, *seo*, *seq *	—	SEB	22	2	− (−0.084)
P16	R	6	*sea*, *seb*, *seg*, *sei*, *sem*,* sen*, *seo*, *seq *	—	SEB	22	2	− (−0.249)
P17	R	6	*seb*, *seg*, *sei*, *sem*,* sen*, *seo*, *seq *	—	SEB	22	2	− (−0.051)
P18	R	6	*seb, seg, sei, sem, sen, seo, seq *	—	SEB	22	2	− (−0.137)
P19	R	6	*sea*, *seb*, *seg*, *sei*, *sem*,* sen*, *seo*, *seq *	—	—	22	2	− (−0.117)
P20	S	9	*sea*,* sek*, *sel*, *seq *	—	—	1	1	+ (1.311)
P21	S	9	*sea*, *sec*, *sel*, *seq *	—	—	2	1	− (−0.173)
P22	S	9	*sea*, *seo*, *seq *	—	—	8	1	+ (0.300)
P23	S	9	*sea*, *sec*, *sel*, *seq *	—	SEA, SEC	9	1	− (−0.204)
P24	S	9	*sea*, *seq *	—	—	9	1	+ (2.210)
P25	S	9	*sea*, *sek*, *seq *	—	—	10	4	+ (0.484)
P26	S	9	*sea*, *sek*, *seo*, *seq *	—	—	17	1	+ (1.156)
P27	S	9	*sea*, *seh*, *sek*, *seq *	—	SEA	17	3	− (−0.058)
P28	S	9	*sea*, *sed*, *sei*, *seq *	*etd *	SED	18	1	− (−0.225)
P29	S	9	*sea*, *seq *	—	—	18	2	+ (0.098)
P30	S	9	*sea*, *sec*, *seg*, *sei*, *sel*, *sem*, *sen*, *seo*, *seq *	—	SEC	18	2	− (−0.390)
P31	S	9	*sea*, *seb*, *seg*, *sei*, *sem*, *sen*, *seo*, *seq*, *seu *	*eta *	—	18	2	− (−0.147)
P32	S	9	*sea*, *sec*, *seg*, *sei*,* sel*, *sen*, *seo*, *seq *	—	SEC	20	3	+ (0.128)
P33	S	9	*seq *	—	ND	20	3	− (−0.245)
P34	S	9	*seo*, *seq *	—	ND	22	1	− (−0.326)
P35	S	9	*sea*, *seo*, *seq *	—	—	23	1	+ (0.054)
P36	S	9	*sed*, *sek*, *seq *	—	ND	23	1	+ (1.107)

T1	S	9	*sea*, *sen*, *seq *	—	—	5	2	− (−0.073)
T2	S	9	*sea*, *seg*, *sem*, *sen*, *seo*,* seq *	—	SEA	5	2	+ (0.046)
T3	S	9	*seb*, *seg*, *sei*, *sem*, *sen*, *seo*, *seq*, *seu *	*eta *	SEB, ETA	8	4	+ (3.872)
T4	S	9	*sea*, *seg*, *sen*, *seq *	—	—	9	2	+ (0.319)
T5	S	9	*sea*, *seg*, *sei*,* sek*, *sem*, *sen*, *seo*,* seq*, *seu *	—	—	9	4	− (−0.081)
T6	S	9	*seg*, *sen*, *seq *	—	ND	11	1	+ (0.736)
T7	S	9	*sea*, *seg*,* sei*,* sek*, *sen*, *seo *	—	—	15	2	+ (2.818)
T8	S	9	*sea*, *seg*, *sei*, *sem*, *sen*, *seo*, *seq *	*eta *	—	18	1	+ (0.156)
T9	S	10	*seq *	*etd *	ND	12	1	− (−0.114)
T10	S	10	*sea*, *seg*, *sei*,* sem*, *sen*, *seo*, *seq *	—	SEA	14	2	+ (0.086)
T11	S	10	*sea*, *seg*, *sei*, *sem*, *sen*, *seo*,* seq *	—	—	15	2	+ (0.808)
T12	S	10	*sec*,* seh*, *sel*, *seq *	—	SEC	15	3	− (−0.198)
T13	S	10	*sea*, *sek*, *seq *	*etd *	SEA	18	1	+ (0.235)

−: not detectable, +: produced biofilm.

ND: not done.
